# Minimum Risk Facility Location-Allocation Problem with Type-2 Fuzzy Variables

**DOI:** 10.1155/2014/472623

**Published:** 2014-03-20

**Authors:** Xuejie Bai, Ying Liu

**Affiliations:** ^1^College of Science, Agricultural University of Hebei, Baoding, Hebei 071001, China; ^2^College of Management, Hebei University, Baoding, Hebei 071002, China

## Abstract

Facility location decision is basically viewed as a long-term strategy, so the inherited uncertainty of main parameters ought to be taken into account in order to make models applicable. In this paper, we examine the impact of uncertain transportation costs and customers' demands on the choice of optimal location decisions and allocation plans. This leads to the formulation of the facility location-allocation (FLA) problem as a fuzzy minimum risk programming, in which the uncertain parameters are assumed to be characterized by type-2 fuzzy variables with known type-2 possibility distributions. Since the inherent complexity of type-2 fuzzy FLA may be troublesome, existing methods are no longer effective in handling the proposed problems directly. We first derive the critical value formula for possibility value-at-risk reduced fuzzy variable of type-2 triangular fuzzy variable. On the basis of formula obtained, we can convert original fuzzy FLA model into its equivalent parametric mixed integer programming form, which can be solved by conventional numerical algorithms or general-purpose software. Taking use of structural characteristics of the equivalent optimization, we design a parameter decomposition method. Finally, a numerical example is presented to highlight the significance of the fuzzy FLA model. The computational results show the credibility and superiority of the proposed parametric optimization method.

## 1. Introduction

Facility location-allocation problem consists of how to choose the optimal location among a given set of possible sites and simultaneously how to arrange the allocation of the available products such that the total cost is minimized. The concept of FLA was initially proposed by Cooper [1]. Since then, FLA has attracted more and more researchers' attention [2–4] and has been successfully applied in many real-world fields such as emergency service systems, telecommunication networks, gas stations, automated teller machines, and supply chain management. Along with a bewildering variety of FLA models, numerous algorithms have been designed such as the branch-and-bound algorithm [5], simulated annealing [6], and tabu search [7]. A thorough coverage of the most FLA variants and a broad overview of their mathematical formulations as well as case studies can be found in the work of Arabani and Farahani [8] and Drezner and Hamacher [9].

Facility location decisions play a critical role in strategic planning for a wide range of private and public firms. The main parameters of models, that is, costs, demands, travel times, and other inputs to classic FLA problem, may be highly uncertain as a result of many factors such as the interaction of customers and suppliers, distribution networks, business climate, and government legislation. The fuzzy programming approach provides a reasonable way to exploit the facility location problem under uncertainty. For example, Liu and Tian [10] designed a hybrid particle swarm optimization algorithm to solve the two-stage fuzzy FLA problem with VaR objective. Shankar et al. [11] proposed a multiobjective location-allocation problem for single-product in four-echelon supply chain architecture and exploited a hybrid algorithm combining the nondominated sorting algorithm and multiobjective particle swarm optimization to solve the model. Wen and Kang [12] considered some FLA models, such as the expected cost minimization model, (*α*, *β*)-cost minimization model, and chance maximization model with random fuzzy demands, and integrated the simplex algorithm, random fuzzy simulations, and genetic algorithm to produce a hybrid intelligent algorithm. Mousavi and Niaki [13] developed three types of fuzzy programming models: fuzzy expected cost programming, the fuzzy *β*-cost minimization model, and the credibility maximization model according to different decision criteria and solved the problems by a hybrid intelligent algorithm.

In a fuzzy decision system, fuzziness usually is characterized by fuzzy sets. In general, fuzzy set requires crisp membership function which cannot be obtained in practical problems. To overcome this difficulty, the type-2 fuzzy set as an extension of an ordinary fuzzy set was introduced by Zadeh [14] in 1975. After that, there are a lot of researchers who study, extend, and apply type-2 fuzzy sets [15–19]. Among them, Z.-Q. Liu and Y.-K. Liu [15] adopted a variable-based approach to depict type-2 fuzzy phenomenon and presented the fuzzy possibility theory which is a generalization of the usual possibility theory. Qin et al. [18] gave the mean value reduction methods for the type-2 fuzzy variables and applied them to model fuzzy data envelopment analysis. Wu and Liu [19] presented the equivalent value reduction methods and employed them to portfolio selection problems. To the best of our knowledge, there is little research for modeling FLA from type-2 fuzziness standpoint. In the current development, we will formulate a new fuzzy FLA model, in which the transportation costs and the customers' demands are characterized by type-2 fuzzy variables. More precisely, the fuzzy costs and demands can be represented by parametric possibility distributions, which are obtained by using the possibility value-at-risk (VaR) reduction method [20]. In order to solve the proposed minimum risk FLA model, we deduce the critical value formula of the reduced fuzzy variables, which are used to turn the original model with service quality constraints into its equivalent parametric mixed integer programming that can be solved by general-purpose software. One numerical experiment is performed for the sake of illustration.

The rest of this paper is organized as follows.  Section 2 derives the critical value formula of the reduced fuzzy variables for common type-2 triangular fuzzy variable. In Section 3, we develop a new fuzzy FLA model with minimum risk criterion. In Section 4, by means of the results obtained, we convert the original FLA problem to its equivalent model. In Section 5, one numerical example is given to highlight the significance of the proposed model and the superiority of parametric method. Finally, Section 6 summarizes the main conclusions in our paper.

## 2. Critical Value Formulas of Reduced Fuzzy Variables

Let *ξ* be a regular fuzzy variable. Then the upper VaR of *ξ* with respect to possibility, denoted by VaR^*U*^(*ξ*), is defined as

(1) $$\begin{array}{c} {\text{VaR}}_{\alpha }^{U}\left(\xi \right)=\sup\left\{x\;|\;\text{Pos}\left\{\xi \geq x\right\}\geq \alpha \right\}, \end{array}$$

while the lower VaR of *ξ* with respect to possibility, denoted by VaR^*L*^(*ξ*), is defined as

(2) $$\begin{array}{c} {\text{VaR}}_{\alpha }^{L}\left(\xi \right)=\inf\left\{\;x\;|\;\text{Pos}\left\{\xi \leq x\right\}\geq \alpha \right\}. \end{array}$$

Let $\left(\Gamma ,𝒜,\tilde{\text{P}}\text{os}\right)$ be a fuzzy possibility space [15] and $\tilde{\xi }$ a type-2 fuzzy variable with secondary possibility distribution ${\tilde{\mu }}_{\tilde{\xi }}(x)$. To reduce the uncertainty in ${\tilde{\mu }}_{\tilde{\xi }}(x)$, we will give a new representation for the regular fuzzy variable ${\tilde{\mu }}_{\tilde{\xi }}(x)$ and employ the lower and upper VaRs of $\tilde{\text{P}}\text{os}\{\gamma \in \Gamma |\tilde{\xi }(\gamma )=x\}$ as the representing values. The method is referred to as the possibility VaR reduction [20]. The variables obtained by the VaR reduction methods are called the lower and upper VaR reduced fuzzy variables and denoted by *ξ* ^*U*^ and *ξ* ^*L*^, respectively.

A type-2 fuzzy variable $\tilde{\xi }$ is called triangular if its secondary possibility distribution ${\tilde{\mu }}_{\tilde{\xi }}(x)$ is

(3) (x−r1r2−r1−θlmin⁡{x−r1r2−r1,r2−xr2−r1},x−r1r2−r1,x−r1r2−r1+θrmin⁡{x−r1r2−r1,r2−xr2−r1}),

for any *x* ∈ [*r*_1_, *r*_2_], and

(4) (r3−xr3−r2−θlmin⁡{r3−xr3−r2,x−r2r3−r2},r3−xr3−r2,r3−xr3−r2+θrmin⁡{r3−xr3−r2,x−r2r3−r2}),

for any *x* ∈ [*r*_2_, *r*_3_], where *θ*_*l*_, *θ*_*r*_ ∈ [0,1] are two parameters characterizing the degree of uncertainty that $\tilde{\xi }$ takes the value *x*. For simplicity, we denote the type-2 triangular fuzzy variable $\tilde{\xi }$ with the distribution above by (*r*_1_, *r*_2_, *r*_3_; *θ*_*l*_, *θ*_*r*_). If we denote *θ* = (*θ*_*l*_, *θ*_*r*_), then the reduced fuzzy variables *ξ* ^*U*^ and *ξ* ^*L*^ have the following parametric possibility distributions:

(5) μξU(x;θ,α) ={(1+θr−αθr)x−r1r2−r1,if  x∈[r1,r1+r22](1−θr+αθr)x+(1−α)θrr2−r1r2−r1,if  x∈[r1+r22,r2]−(1−θr+αθr)x−(1−α)θrr2+r3r3−r2,if  x∈[r2,r2+r32](1+θr−αθr)r3−xr3−r2,if  x∈[r2+r32,r3],

(6) μξL(x;θ,α)={(1−θl+αθl)x−r1r2−r1,if  x∈[r1,r1+r22](1+θl−αθl)x−(1−α)θlr2−r1r2−r1,if  x∈[r1+r22,r2]−(1+θl−αθl)x+(1−α)θlr2+r3r3−r2,if  x∈[r2,r2+r32](1−θl+αθl)r3−xr3−r2,if  x∈[r2+r32,r3].

**Theorem 1**

Let $\tilde{\xi }=(r_{1},r_{2},r_{3};\theta_{l},\theta_{r})$ be a type-2 triangular fuzzy variable. If we denote *θ* = (*θ*_*l*_, *θ*_*r*_), then the critical values of the upper reduced fuzzy variable *ξ* ^*U*^ have the following parametric possibility distributions:

(7) ξinf⁡U(β;θ,α) ={r1+2β(r2−r1)1+θr−αθr,if  β∈[0,1+θr−αθr4]r2−(1−2β)(r2−r1)1−θr+αθr,if  β∈[1+θr−αθr4,12]r2+(2β−1)(r3−r2)1−θr+αθr,if  β∈[12,3−θr+αθr4]r3−2(1−β)(r3−r2)1+θr−αθr,if  β∈[3−θr+αθr4,1];ξsup⁡U(β;θ,α) ={r3−2β(r3−r2)1+θr−αθr,if  β∈[0,1+θr−αθr4]r2+(1−2β)(r3−r2)1−θr+αθr,if  β∈[1+θr−αθr4,12]r2−(2β−1)(r2−r1)1−θr+αθr,if  β∈[12,3−θr+αθr4]r1+2(1−β)(r2−r1)1+θr−αθr,if  β∈[3−θr+αθr4,1].

**Proof**

We only prove the first equation, and the second one can be proved similarly.

Since *ξ* ^*U*^ is the upper reduced fuzzy variable of $\tilde{\xi }$, its parametric possibility distribution *μ*_*ξ*^*U*^_(*x*) is given by (5). On the basis of the definition of the pessimistic value of fuzzy variables, we have

(8) $$\begin{array}{c} \xi_{\inf}^{U}\left(\beta ;\theta ,\alpha \right)=\inf\left\{x\;|\;\text{Cr}\left\{\xi^{U}\leq x\right\}\geq \beta \right\}. \end{array}$$

According to the parametric possibility distribution *μ*_*ξ*^*U*^_(*x*), we have

(9) Cr{ξU≤x} ={0, if  x∈(−∞,r1](1+θr−αθr)x−r12(r2−r1),if  x∈[r1,r1+r22](1−θr+αθr)x+(1−α)θrr2−r12(r2−r1),if  x∈[r1+r22,r2]1−−(1−θr+αθr)x−(1−α)θrr2+r32(r3−r2),if  x∈[r2,r2+r32]1−(1+θr−αθr)r3−x2(r3−r2),if  x∈[r2+r32,r3],1, if  x∈[r3,+∞).

Note that *μ*_*ξ*^*U*^_((*r*_1_ + *r*_2_)/2) = (1 + *θ*_*r*_ − *αθ*_*r*_)/4 and *μ*_*ξ*^*U*^_((*r*_2_ + *r*_3_)/2) = (3 − *θ*_*r*_ + *αθ*_*r*_)/4.

When *β* ∈ (0, (1 + *θ*_*r*_ − *αθ*_*r*_)/4), *ξ*_inf⁡_^*U*^(*β*; *θ*, *α*) is the solution of the following equation:

(10) $$\begin{array}{c} \left(1+\theta_{r}-\alpha \theta_{r}\right)\frac{x-r_{1}}{2\left(r_{2}-r_{1}\right)}=\beta . \end{array}$$

By solving the above equation, we have

(11) $$\begin{array}{c} \xi_{\inf}^{U}\left(\beta ;\theta ,\alpha \right)=r_{1}+\frac{2\beta \left(r_{2}-r_{1}\right)}{1+\theta_{r}-\alpha \theta_{r}}. \end{array}$$

As for *β* ∈ ((1 + *θ*_*r*_ − *αθ*_*r*_)/4,1/2), (1/2, (3 − *θ*_*r*_ + *αθ*_*r*_)/4) and ((3 − *θ*_*r*_ + *αθ*_*r*_)/4,1), it is similar to deduce

(12) $$\begin{array}{c} \xi_{\inf}^{U}\left(\beta ;\theta ,\alpha \right)=r_{2}-\frac{\left(1-2\beta \right)\left(r_{2}-r_{1}\right)}{1-\theta_{r}+\alpha \theta_{r}},\\ \xi_{\inf}^{U}\left(\beta ;\theta ,\alpha \right)=r_{2}+\frac{\left(2\beta -1\right)\left(r_{3}-r_{2}\right)}{1-\theta_{r}+\alpha \theta_{r}}, \end{array}$$

and

(13) $$\begin{array}{c} \xi_{\inf}^{U}\left(\beta ;\theta ,\alpha \right)=r_{3}-\frac{2\left(1-\beta \right)\left(r_{3}-r_{2}\right)}{1+\theta_{r}-\alpha \theta_{r}}, \end{array}$$

respectively.

Hence, we have

(14) ξinf⁡U(β;θ,α) ={r1+2β(r2−r1)1+θr−αθr,if  β∈[0,1+θr−αθr4]r2−(1−2β)(r2−r1)1−θr+αθr,if  β∈[1+θr−αθr4,12]r2+(2β−1)(r3−r2)1−θr+αθr,if  β∈[12,3−θr+αθr4]r3−2(1−β)(r3−r2)1+θr−αθr,if  β∈[3−θr+αθr4,1].

The proof of the assertion is complete.

**Theorem 2**

Let $\tilde{\xi }=(r_{1},r_{2},r_{3};\theta_{l},\theta_{r})$ be a type-2 triangular fuzzy variable. If we denote *θ* = (*θ*_*l*_, *θ*_*r*_), then the critical values of the lower reduced fuzzy variable *ξ* ^*L*^ have the following parametric possibility distributions:

(15) ξinf⁡L(β;θ,α) ={r1+2β(r2−r1)1−θl+αθl,if  β∈[0,1−θl+αθl4]r2−(1−2β)(r2−r1)1+θl−αθl,if  β∈[1−θl+αθl4,12]r2+(2β−1)(r3−r2)1+θl−αθl,if  β∈[12,3+θl−αθl4]r3−2(1−β)(r3−r2)1−θl+αθl,if  β∈[3+θl−αθl4,1];ξsup⁡L(β;θ,α) ={r3−2β(r3−r2)1−θl+αθl,if  β∈[0,1−θl+αθl4]r2+(1−2β)(r3−r2)1+θl−αθl,if  β∈[1−θl+αθl4,12]r2−(2β−1)(r2−r1)1+θl−αθl,if  β∈[12,3+θl−αθl4]r1+2(1−β)(r2−r1)1−θl+αθl,if  β∈[3+θl−αθl4,1].

**Proof**

It can be proved similarly as Theorem 1.

The following corollaries show that the critical values of the VaR-based reduced fuzzy variables extend that of the expectation-based reduced fuzzy variables [18] for the type-2 triangular fuzzy variable.

**Corollary 3**

Let $\tilde{\xi }$ be a type-2 triangular fuzzy variable and let *ξ* ^1^, *ξ* ^2^, and *ξ* ^3^ be the reduced fuzzy variables obtained by **E**_∗_, E*, and **E** reduction method, respectively.

- For **E**_∗_ reduction method, *ξ*_sup⁡_^*L*^(*β*; *θ*, (1/2)) = *ξ*_sup⁡_^1^(*β*);
- For E* reduction method, *ξ*_sup⁡_^*U*^(*β*; *θ*, (1/2)) = *ξ*_sup⁡_^2^(*β*);
- For **E** reduction method, we have
  - If *θ*_*l*_ ≥ *θ*_*r*_, then *ξ*_sup⁡_^*L*^(*β*; *θ*, ((3*θ*_*l*_ + *θ*_*r*_)/4*θ*_*l*_)) = *ξ*_sup⁡_^3^(*β*);
  - If *θ*_*l*_ ≤ *θ*_*r*_, then *ξ*_sup⁡_^*U*^(*β*; *θ*, ((*θ*_*l*_ + 3*θ*_*r*_)/4*θ*_*r*_)) = *ξ*_sup⁡_^3^(*β*),

where *ξ*_sup⁡_^1^(*β*), *ξ*_sup⁡_^2^(*β*), and *ξ*_sup⁡_^3^(*β*) are expressed in [21].

The results mentioned above imply that the new method is much more robust to implement than the existing methods when we employ it to build a mathematical model with type-2 fuzzy coefficients.

**Remark 4**

For the pessimistic values of reduced fuzzy variables obtained by either possibility VaR-based or expectation-based reduction methods, we have similar results.

## 3. Formulation of Fuzzy FLA Model

Facility location-allocation problem was first proposed by Cooper [1] to study the problem of how to locate a set of new facilities to satisfy the customers' demands so that the total costs of opening facilities and variable operating cost are minimized. In the past, the parameters in the FLA model were known precisely. However, in many cases, the data cannot be known with certainty. The uncertainty in costs associated with transportation of final products may be caused by traffic congestion, weather conditions, fuel price fluctuations, and so on. Additionally, the customers' demands are also subject to uncertainty due to economic instability and market fluctuations besides other endogenous and exogenous factors. In this paper, we will develop a robust approach to dealing with fuzzy FLA problem. In our method, we will employ parametric possibility distribution functions instead of fixed possibility distribution functions to describe the uncertain parameters, and the parametric possibility distributions are obtained by using the possibility VaR reduction method. That is to say, the reduced fuzzy demands and costs have parametric possibility distributions, so they can serve as the representatives of type-2 customers' demands and transportation costs. In the following, we will adopt this modeling idea to construct fuzzy FLA problem. In the interest of brevity, we will display the parameters in Abbreviations Section.

Based on the notations above, the FLA model can be given as follows:

(16) min⁡ ∑i=1mfixi+∑i=1m∑j=1nξ~ijxijs.t. ∑j=1nxij≤Mxi, i=1,2,…,m∑i=1mxij≥d~j j=1,2,…,nxi∈{0,1} i=1,2,…,mxij≥0 i=1,2,…,m; j=1,2,…,n.

In this situation, the objective value of model (16) is also a type-2 fuzzy variable, but it is meaningless to minimize the type-2 fuzzy variable without giving any criteria in advance. At the same time, the meaning of the constraints of model (16) is not clear, so we cannot judge whether or not a decision vector is feasible. Therefore, the form (16) is not well defined mathematically. To build a meaningful model, we can employ the possibility VaR reduction method to simplify the type-2 fuzzy variables ${\tilde{\xi }}_{ij}$ and ${\tilde{d}}_{j}$ in the model (16) so as to get their reduced fuzzy variables *ξ*_*ij*_^*U*^ and *d*_*j*_^*L*^. If a decision maker wants to obtain a decision with minimum risk, then a new class of fuzzy FLA model may be constructed:

(17) max⁡ Cr{∑i=1mfixi+∑i=1m∑j=1nξijUxij≤c0}s.t. ∑j=1nxij≤Mxi i=1,2,…,mCr{∑i=1mxij≥djL}≥βj j=1,2,…,nxi∈{0,1}, xij≥0 i=1,2,…,m;j=1,2,…,n.

The goal of fuzzy facility location-allocation model (17) is to choose at which location to open facilities and how to assign the commodities from facilities to customers such that the credibility is maximized that the total expected cost of opening and operating facilities do not exceed some given value *c*_0_. The first constraint makes certain that the products are assigned to open facilities and that the distribution amounts do not exceed the facility capacity. In principle, the firm expects to satisfy the demands of customers exactly. However, in the real world, many unforeseen events will cause the change of the customers' demands. The second constraint represents that the distribution amounts from different facilities to customer *j* should meet the customer's demand with a given service lever *β*_*j*_. The rest of the constraints are for the binary and nonnegativity restrictions.

With additional variable *β*, model (17) is equivalent to the following mathematical programming model with a number of credibility constraints:

(18) max⁡ βs.t. Cr{∑i=1mfixi+∑i=1m∑j=1nξijUxij≤c0}≥β∑j=1nxij≤Mxi i=1,2,…,mCr{∑i=1mxij≥djL}≥βj j=1,2,…,nxi∈{0,1}, xij≥0 i=1,2,…,m; j=1,2,…,n.

In order to solve the fuzzy FLA model presented, it is required to compute the credibility of fuzzy events in the objective and in the constraints. In the next section, we discuss some special cases, where the uncertain parameters are characterized by independent type-2 triangular fuzzy variables.

## 4. Equivalent Representation of Fuzzy FLA Model

In our fuzzy FLA model, the parameter ${\tilde{\xi }}_{ij}$ means the transportation cost from facility *i* to customer *j*. The transportation costs ${\tilde{\xi }}_{ij}$ are different for every *i* and *j*, but they are affected by some common factors. So we introduce type-2 fuzzy variable ${\tilde{\xi }}_{i}$ that can be seen as basic transportation cost and can rewrite ${\tilde{\xi }}_{ij}$ as a simple function of ${\tilde{\xi }}_{i}$, that is, ${\tilde{\xi }}_{ij}=c_{ij}{\tilde{\xi }}_{i}$, where *c*_*ij*_ is real number and comes from the interval [1.5,2.5] randomly.

Hence, all the type-2 fuzzy variables in the objective turn into the functions of ${\tilde{\xi }}_{1},{\tilde{\xi }}_{2},\ldots ,{\tilde{\xi }}_{m}$, together with the type-2 fuzzy variables ${\tilde{d}}_{1},{\tilde{d}}_{2},\ldots ,{\tilde{d}}_{n}$ in the service level constraints, we only need to deal with the *m* + *n* type-2 fuzzy variables. Assume that ${\tilde{\xi }}_{i}$ and ${\tilde{d}}_{j}$ are mutually independent type-2 triangular fuzzy variables such that their elements are defined by

(19) $$\begin{array}{c} {\tilde{\xi }}_{i}=\left(r_{1,i},r_{2,i},r_{3,i};\theta_{l,i},\theta_{r,i}\right),\\ {\tilde{d}}_{j}=\left(r_{1,j},r_{2,j},r_{3,j};{\bar{\theta }}_{l,j},{\bar{\theta }}_{r,j}\right). \end{array}$$

Suppose that *ξ*_*i*_^*U*^ and *d*_*j*_^*L*^ are the reduced fuzzy variables of ${\tilde{\xi }}_{i}$ and ${\tilde{d}}_{j}$, respectively. Obviously, *ξ*_*i*_^*U*^ and *d*_*j*_^*L*^ are mutually independent fuzzy variables. Thus, the total cost constraint Cr{∑_*i*=1_^*m*^ *f*_*i*_ *x*_*i*_ + ∑_*i*=1_^*m*^∑_*j*=1_^*n*^ *ξ*_*ij*_^*U*^ *x*_*ij*_ ≤ *c*_0_} ≥ *β* has the following equivalent expression:

(20) c0−∑i=1mfixi≥(∑i=1m∑j=1nξijUxij)inf⁡(β)=∑i=1m∑j=1nξij,inf⁡U(β)xij=∑i=1m∑j=1ncijξi,inf⁡U(β)xij.

For the sake of description, we take *β* to be more than 0.5. Let *θ*_*l*_ = max⁡{*θ*_*l*,*i*_} and *θ*_*r*_ = min⁡{*θ*_*r*,*i*_}. Then, on the basis of Theorem 3 [22] and Theorem 1, if 0.5 < *β* ≤ (3 − (1 − *α* ^*U*^)*θ*_*r*_)/4, (20) is equivalent to

(21) ∑i=1mfixi+∑i=1m∑j=1ncij(r2,i+(2β−1)(r3,i−r2,i)1−θr+αUθr)xij≤c0.

If not, (20) is equivalent to

(22) ∑i=1mfixi  +∑i=1m∑j=1ncij(r3,i−2(1−β)(r3,i−r2,i)1+θr−αUθr)xij≤c0.

Similarly, consider the fuzzy demand *d*_*j*_^*L*^ in the service level constraint. We find that Cr{∑_*i*=1_^*m*^ *x*_*ij*_ ≥ *d*_*j*_^*L*^} ≥ *β*_*j*_ has the following equivalent expression:

(23) $$\begin{array}{c} \overset{m}{\underset{i=1}{\sum }}x_{ij}\geq d_{j,\inf}\left(\beta_{j}\right). \end{array}$$

Let *β*_*j*_ > 0.5 and $B=\{j|0.5<\beta_{j}\leq (3+(1-\alpha_{j}^{L}){\bar{\theta }}_{l,j})/4\}$. Then, on the basis of Theorem 2, (23) is equivalent to

(24) $$\begin{array}{c} \overset{m}{\underset{i=1}{\sum }}x_{ij}\geq r_{2,j}+\frac{\left(2\beta_{j}-1\right)\left(r_{3,j}-r_{2,j}\right)}{1+{\bar{\theta }}_{l,j}-\alpha_{j}^{L}{\bar{\theta }}_{l,j}},\;\text{for}\;j\in B, \end{array}$$

or

(25) $$\begin{array}{c} \overset{m}{\underset{i=1}{\sum }}x_{ij}\geq r_{3,j}-\frac{2\left(1-\beta_{j}\right)\left(r_{3,j}-r_{2,j}\right)}{1-{\bar{\theta }}_{l,j}+\alpha_{j}^{L}{\bar{\theta }}_{l,j}},\;\text{for}\;j\notin B. \end{array}$$

In view of the discussion above, when the uncertain variables are mutually independent type-2 triangular distributions, the exact analytical expressions (21), (22), (24), and (25) of the total cost and service constraints are available. Finally, we can reformulate the equivalent model of fuzzy FLA in the following two forms:

(26) max⁡ βs.t. ∑i=1mfixi+∑i=1m∑j=1ncij(r2,i+(2β−1)(r3,i−r2,i)1−θr+αUθr)xij≤c0∑j=1nxij≤Mxi i=1,2,…,m∑i=1mxij≥r2,j+(2βj−1)(r3,j−r2,j)1+θ¯l,j−αjLθ¯l,j j∈B∑i=1mxij≥r3,j−2(1−βj)(r3,j−r2,j)1−θ¯l,j+αjLθ¯l,j j∉Bxi∈{0,1}, xij≥0 i=1,2,…,m;j=1,2,…,n,

or

(27) max⁡ βs.t. ∑i=1mfixi+∑i=1m∑j=1ncij(r3,i−2(1−β)(r3,i−r2,i)1+θr−αUθr)xij≤c0∑j=1nxij≤Mxi i=1,2,…,m∑i=1mxij≥r2,j+(2βj−1)(r3,j−r2,j)1+θ¯l,j−αjLθ¯l,j j∈B∑i=1mxij≥r3,j−2(1−βj)(r3,j−r2,j)1−θ¯l,j+αjLθ¯l,j j∉Bxi∈{0,1}, xij≥0 i=1,2,…,m;j=1,2,…,n.

For a given confidence level *β*_*j*_, the solution process can be divided into at most two steps by decomposing the feasible region of original model, which is described as follows.

**Step 1**

Solve the mixed-integer programming subproblems (26)-(27), respectively.

**Step 2**

Chose the maximum value as the global optimal value to original model by comparing the optimal values to the two subproblems.

Since the parametric domain of variable *β* is separated into two subregions according to the values of parameter *β*, the solution process is executed at most two times by solving two different subproblems of problem (18). We refer to this approach as the parametric decomposition method.

The models (26) and (27) are the parameter-based mixed integer programming, which can be solved by some conventional algorithms, such as branch-and-bound method. It is known that the LINGO is a state-of-the-art commercial software package including the branch-and-bound IP code.

## 5. One Numerical Example

In this section, we propose an example to demonstrate the modeling idea. The example is described as follows.

In the telemarketing industry, a big firm has confronted many facility location problems in possible sites for the call centers. Such unit calling cost changes dramatically depending on the location of call origin and receiving center; site selection is very important. Suppose that there are 14 customer zones and 8 sites under consideration for Tmark's catalog order centers. Any Tmark center selected can handle at most 2000 call units per day.

This problem was considered in [23] in which the unit calling charges and customers' demands were assumed to be constants. In this paper, we generalize the problem by assuming that the unit calling charges and customers' demands are characterized by mutually type-2 triangular fuzzy variables with known type-2 possibility distributions. The coefficient *c*_*ij*_ is real number and come from the interval [1.5,2.5] randomly. Tables 1, 2, and 3 show the data for our problem.

When *α* ^*U*^ = 0.8, *α*_*j*_^*L*^ = 0.85, *β*_*j*_ = 0.9, and *c*_0_ = 38352, we use domain decomposition method to find the solution of the model (18). For the subproblem (26), we can obtain the local optimal value 0.78097, while for the subproblem (27), we can obtain the local optimal value 0.78688. Consequently, by comparison, we can have the optimal objective value 0.78688 with the optimal solution as follows:

(28) $$\begin{array}{c} x_{1}=1,\;x_{13}=1154.5,\;x_{111}=845.5;\\ x_{4}=1,\;x_{48}=113.5,\;x_{49}=496.9,\\ x_{411}=133.2,\;x_{412}=1256.4;\\ x_{5}=1,\;x_{56}=877.3,\;x_{510}=259,\;x_{513}=453.1;\\ x_{8}=1,\;x_{81}=273.5,\;x_{82}=173.7,\\ x_{84}=85.5,\;x_{85}=79.6,\;x_{87}=337.8,\\ x_{812}=322,\;x_{814}=238.2. \end{array}$$

The optimal solution means that the company needs to open 4 facilities located in 1, 4, 5, and 8. The customer zones 1, 2, 4, 5, 7, and 14 are served only by the facility 8. The customer zone 3 is served by the facility 1. The customer zones 6, 10, and 13 are served only by the facility 5. The customer zones 8 and 9 are served only by the facility 4. The customer zone 11 is served by both the facilities 1 and 4. The customer zone 12 is served by both the facilities 4 and 8, respectively.

**Figure 1:**
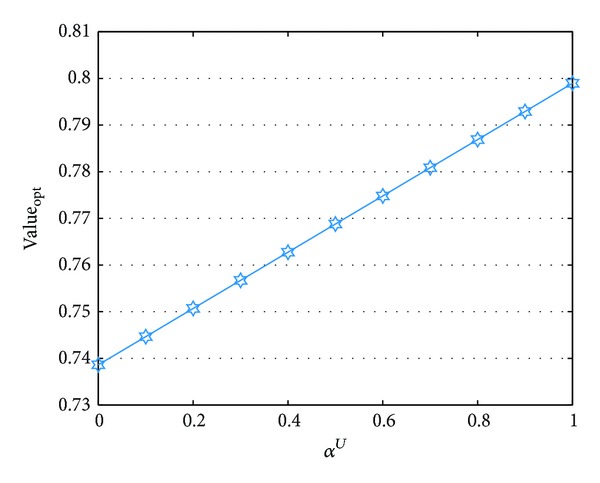
Parameter analysis of *α* ^*U*^.

**Figure 2:**
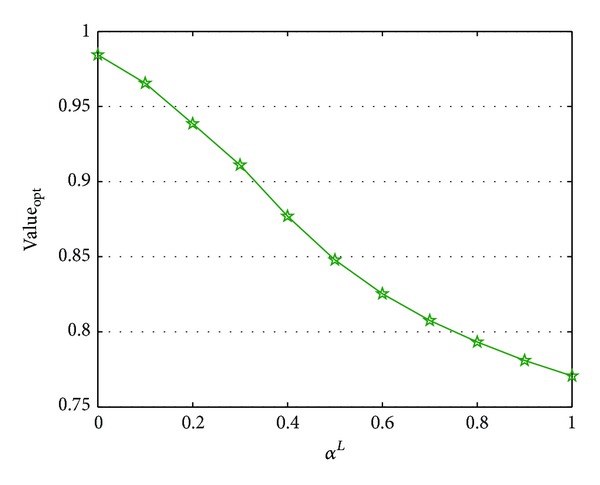
Parameter analysis of *α* ^*L*^.

In order to investigate the parameters' influence on the solution quality, we can compute the optimal value by adjusting slightly the parameters of FLA model. For simplicity, we assume that all *α*_*j*_^*L*^ are equal, denoted as *α* ^*L*^. When the parameters *α* ^*U*^ and *α* ^*L*^ increase with fixed step 0.1 from 0 to 1 assuring other parameters being unchanged, the computational results of fuzzy FLA model corresponding to various different parameters *α* ^*U*^ and *α* ^*L*^ are reported in Figures 1 and 2, where the symbol “Value_opt_” represses the optimal value.

From Figures 1 and 2, we can see that the optimal cost varies while the parameters change. Specifically, the optimal value is a monotone increasing function with respect to *α* ^*U*^ ∈ [0,1] and a monotone decreasing function with respect to *α* ^*L*^ ∈ [0,1]. Therefore, with the method proposed in this paper, the decision maker can make better decisions.

## 6. Conclusions

The facility location-allocation problem is one of the most comprehensive strategic decision issues that need to be optimized for the long-term efficient operation of the firm. The paper extended the traditional FLA model and developed a new fuzzy FLA model with type-2 fuzzy parameters. To summarize, the major distinguishing features of the current research are as follows.

- Theorems 1 and 2 present the critical value formula for the VaR reduced fuzzy variables of type-2 triangular fuzzy variable. Using the formulas, we can reduce the complexity of computing the credibility constraints so that much time can be saved when solving the proposed FLA model.
- For the first time, we proposed a new fuzzy minimum risk facility location-allocation model, in which the unit transportation cost and demands of customers were uncertain and assumed to be type-2 fuzzy variables. On the basis of the possibility VaR reduction method and obtained formula, we converted the original optimization problem into its equivalent parametric programming model and found the corresponding optimal solutions through parametric decomposition method.
- We provided a numerical example to demonstrate the effectiveness of the proposed model. The computational results showed that the parametric method was robust for parameters selection and had advantages for FLA problem.

In a word, this paper studied fuzzy FLA problem from the theoretical and computational viewpoint. The methodologies used in this paper were quite general and can be applied to the decision making problems in different areas with type-2 fuzzy parameters.

## Figures and Tables

**Table 1:** The fixed costs and calling charges for the type-2 fuzzy FLA problem.

Facility	1	2	3	4
*f*_*i*_	2400	7000	3600	1600
${\tilde{\xi }}_{i}$	(2.21,2.32,2.45; θ_*l*,1_, θ_*r*,1_)	(2.47,2.82,2.96; θ_*l*,2_, θ_*r*,2_)	(2.37,2.45,2.50; θ_*l*,3_, θ_*r*,3_)	(2.19,2.27,2.32; θ_*l*,4_, θ_*r*,4_)

Facility	5	6	7	8

*f*_*i*_	3000	4600	9000	2000
${\tilde{\xi }}_{i}$	(1.86,2.33,2.63; θ_*l*,5_, θ_*r*,5_)	(2.25,2.57,2.85; θ_*l*,6_, θ_*r*,6_)	(2.49,2.56,2.89; θ_*l*,7_, θ_*r*,7_)	(1.91,2.12,2.52; θ_*l*,8_, θ_*r*,8_)

**Table 2:** The customers' demands for type-2 fuzzy FLA problem.

Customer	1	2	3
${\tilde{d}}_{j}$	$\left(240,250,280;{\bar{\theta }}_{l,1},{\bar{\theta }}_{r,1}\right)$	$\left(130,150,180;{\bar{\theta }}_{l,2},{\bar{\theta }}_{r,2}\right)$	$\left(800,1000,1200;{\bar{\theta }}_{l,3},{\bar{\theta }}_{r,3}\right)$

Customer	4	5	6

${\tilde{d}}_{j}$	$\left(60,70,90;{\bar{\theta }}_{l,4},{\bar{\theta }}_{r,4}\right)$	$\left(45,60,85;{\bar{\theta }}_{l,5},{\bar{\theta }}_{r,5}\right)$	$\left(700,800,900;{\bar{\theta }}_{l,6},{\bar{\theta }}_{r,6}\right)$

Customer	7	8	9

${\tilde{d}}_{j}$	$\left(300,330,340;{\bar{\theta }}_{l,7},{\bar{\theta }}_{r,7}\right)$	$\left(80,90,120;{\bar{\theta }}_{l,8},{\bar{\theta }}_{r,8}\right)$	$\left(450,485,500;{\bar{\theta }}_{l,9},{\bar{\theta }}_{r,9}\right)$

Customer	10	11	12

${\tilde{d}}_{j}$	$\left(200,220,270;{\bar{\theta }}_{l,10},{\bar{\theta }}_{r,10}\right)$	$\left(800,900,1000;{\bar{\theta }}_{l,11},{\bar{\theta }}_{r,11}\right)$	$\left(1400,1500,1600;{\bar{\theta }}_{l,12},{\bar{\theta }}_{r,12}\right)$

Customer	13	14	

${\tilde{d}}_{j}$	$\left(400,430,460;{\bar{\theta }}_{l,13},{\bar{\theta }}_{r,13}\right)$	$\left(180,200,250;{\bar{\theta }}_{l,14},{\bar{\theta }}_{r,14}\right)$	

**Table 3:** The parameters for type-2 triangular fuzzy variables.

*i*	Facility													
	1.	2.	3.	4.	5.	6.	7.	8.						
θ_*l*,*i*_	0.9	0.3	0.2	0.6	0.9	0.5	0.4	0.3						
θ_*r*,*i*_	0.8	0.7	0.4	0.5	0.6	0.3	0.9	0.6						

*j*	Customer													
	1.	2.	3.	4.	5.	6.	7.	8.	9.	10.	11.	12.	13.	14.

${\bar{\theta }}_{l,j}$	0.5	0.3	0.8	0.7	0.5	0.8	0.6	0.5	0.3	0.6	0.4	0.5	0.9	1.0
${\bar{\theta }}_{r,j}$	0.5	0.4	0.4	0.9	0.6	0.1	0.3	0.9	0.7	0.2	0.8	0.2	0.6	0.8

## Abbreviations

*i*: Index of facilities, *i* = 1,2,…, *m*
*j*: Index of customers, *j* = 1,2,…, *n*
*f*_*i*_: The nonnegative fixed cost for opening the facility *i*
*M*: The maximum possible capacity of a facility
${\tilde{\xi }}_{ij}$: The type-2 fuzzy cost used to satisfy the demand of customer *j* from facility *i*
${\tilde{d}}_{j}$: The type-2 fuzzy demand of customer *j*
*x*_*i*_: Binary variable indicating whether facility *i* is open or not
*x*_*ij*_: The amount transported by facility *i* for customer *j*.

## References

[1] Cooper L (1963). Location-allocation problems. *Operations Research*.

[2] Badri MA (1999). Combining the analytic hierarchy process and goal programming for global facility location-allocation problem. *International Journal of Production Economics*.

[3] Hakimi SL (1964). Optimum locations of switching centers and the absolute centers and medians of a graph. *Operations Research*.

[4] Lee SM, Green GI, Kim CS (1981). A multiple criteria model for the location-allocation problem. *Computers & Operations Research*.

[5] Kuenne RE, Soland RM (1972). Exact and approximate solutions to the multisource weber problem. *Mathematical Programming*.

[6] Murray AT, Church RL (1996). Applying simulated annealing to location-planning models. *Journal of Heuristics*.

[7] Ohlemüller M (1997). Tabu search for large location-allocation problems. *The Journal of the Operational Research Society*.

[8] Arabani AB, Farahani RZ (2012). Facility location dynamics: an overview of classifications and applications. *Computers & Industrial Engineering*.

[9] Drezner Z, Hamacher HW (2004). *Facility Location: Applications and Theory*.

[10] Liu Y-K, Tian M (2009). Convergence of optimal solutions about approximation scheme for fuzzy programming with minimum-risk criteria. *Computers & Mathematics with Applications*.

[11] Shankar BL, Basavarajappa S, Chen JCH, Kadadevaramath RS (2013). Location and allocation decisions for multi-echelon supply chain network—a multi-objective evolutionary approach. *Expert Systems with Applications*.

[12] Wen M, Kang R (2011). Some optimal models for facility location-allocation problem with random fuzzy demands. *Applied Soft Computing*.

[13] Mousavi SM, Niaki STA (2013). Capacitated location allocation problem with stochastic location and fuzzy demand: a hybrid algorithm. *Applied Mathematical Modelling*.

[14] Zadeh LA (1975). The concept of a linguistic variable and its application to approximate reasoning—I. *Information Sciences*.

[15] Liu Z-Q, Liu Y-K (2010). Type-2 fuzzy variables and their arithmetic. *Soft Computing*.

[16] Mendel JM, John RIB (2002). Type-2 fuzzy sets made simple. *IEEE Transactions on Fuzzy Systems*.

[17] Mendel JM, John RI, Liu F (2006). Interval type-2 fuzzy logic systems made simple. *IEEE Transactions on Fuzzy Systems*.

[18] Qin R, Liu Y, Liu Z-Q (2011). Modeling fuzzy data envelopment analysis by parametric programming method. *Expert Systems with Applications*.

[19] Wu X-L, Liu Y-K (2012). Optimizing fuzzy portfolio selection problems by parametric quadratic programming. *Fuzzy Optimization and Decision Making*.

[20] Bai X, Liu Y (2013). Semideviations of reduced fuzzy variables: a possibility approach. *Fuzzy Optimization and Decision Making*.

[21] Chen Y, Liu Y (2012). Value-at-risk criteria for uncertain portfolio optimization problem with minimum regret. *Journal of Uncertain Systems*.

[22] Liu Y, Bai X (2014). Linear combinations of T2 fuzzy variables. *Journal of Uncertain Systems*.

[23] Rardin RL (2007). *Optimization in Operation Research*.

